# Intersectional inequalities in COVID-19 morbidity and mortality in Sweden: a retrospective population-based cohort study

**DOI:** 10.1136/bmjph-2025-004719

**Published:** 2026-04-10

**Authors:** Helena Honkaniemi, Sol P Juárez

**Affiliations:** 1 Department of Public Health Sciences, Stockholm University, Stockholm, Sweden; 2 Centre for Health Equity Studies, Stockholm, Sweden; 3 Department of Global Public Health, Karolinska Institutet, Stockholm, Sweden

**Keywords:** COVID-19, Epidemiology, Public Health, Social Medicine

## Abstract

**Introduction:**

During the global pandemic, the elderly, foreign-born and socioeconomically deprived experienced disproportionately higher rates of COVID-19 morbidity and mortality than general populations. However, these groups have typically been analysed in isolation, without considering how social factors jointly influenced COVID-19-related health. Thus, the aim of this study is to quantify intersectional inequalities in COVID-19 morbidity and mortality in Sweden.

**Methods:**

In this retrospective cohort study, national registers were used to identify 35-to-100-year-old residents of Sweden in January 2020 (n=6 014 164), tracking COVID-19 hospitalisations and deaths (International Classification of Diseases-10 Codes U07.1/U07.2, plus B34.2 for deaths) until December 2022. Intersectional multilevel analysis of individual heterogeneity and discriminatory accuracy was applied with logistic regression, nesting individuals within intersectional strata based on age, gender, migrant status and region of origin, and income. Measures of discriminatory accuracy (area under the receiver operating characteristic curve or AUC), predicted probabilities and random effects were calculated.

**Results:**

During follow-up, 77 844 (1.29%) individuals were hospitalised and 18 126 (0.30%) died due to COVID-19. Intersectional models explained 2.22% of the variation in mortality (AUC=0.835) vs 0.60% in hospitalisations (AUC=0.728). Predicted probabilities were lowest in 35–44-year-old Swedish-born women with high income at 0.19% (95% CI 0.16% to 0.21%) for hospitalisations, and 0.01% (95% CI 0.01% to 0.02%) for mortality; and highest among 85–100-year-old foreign-born men from the Global South with low income at 14.85% (95% CI 12.84% to 17.10%) for hospitalisations, and 8.25% (95% CI 6.32% to 10.70%) for mortality. Random effects indicated wider income inequalities in both outcomes in the youngest and narrower in the oldest, native-born and foreign-born groups from the Global North.

**Conclusions:**

Intersectional inequalities in COVID-19 outcomes consistently increased with age, decreased with income and were higher for men and foreign-born individuals. The results emphasise the need to consider universal public health strategies to reach vulnerable populations during a pandemic.

**WHAT IS ALREADY KNOWN ON THIS TOPIC**

- Vulnerable groups including the elderly, migrants and those with low socioeconomic status had higher risks of severe COVID-19 disease and death during the pandemic.
- Most research has examined these groups in isolation without considering how intersecting social categories affected COVID-19 outcomes.

**WHAT THIS STUDY ADDS**

- Probabilities of COVID-19 disease and death were lowest among young, high-income, native-born women and highest among the oldest, low-income, foreign-born men from the Global South.
- Income inequalities in COVID-19 risks were wider in working-age groups than in the elderly.

**HOW THIS STUDY MIGHT AFFECT RESEARCH, PRACTICE OR POLICY**

- Universal prevention measures should be implemented during a pandemic to protect vulnerable groups that are otherwise difficult to identify or reach in a public health crisis.

## Introduction

 Half a decade after the onset of the COVID-19 pandemic, there is ample evidence of its unequal effects on vulnerable groups, dispelling the myth that the pandemic did not ‘discriminate’.^1^ The elderly,^2^ racial and ethnic minorities as well as migrants,^3 4^ and groups with low socioeconomic status^5^ were all disproportionately affected from its early stages, with higher risks of SARS-CoV-2 infection as well as severe COVID-19-related disease and death relative to general populations. Not only did the pandemic reinforce and amplify pre-existing health inequalities among these groups, but it possibly generated new ones.^6^ As an example, international migrants experienced COVID-19-related risks on par with other socially vulnerable populations in many countries, including in Sweden,^3^ despite their pre-pandemic advantages in related health dimensions.^7 8^

To understand the unequal health impacts of the pandemic, most research has either focused on COVID-19 outcomes within specific vulnerable populations (such as racial and ethnic minorities and migrants) or across various social determinants of health (such as income, housing and employment) within the general population.^2^,^5^ However, these subpopulations and social factors have typically been analysed in isolation. In order to move beyond this fragmented approach in research on health inequalities in general^9^ and COVID-19 specifically,^10 11^ there have been calls to adopt an intersectionality perspective by acknowledging that multiple forms of social stratification can interact to shape people’s health in complex and distinctive ways. Failure to recognise how systems of inequality jointly influence health may not only result in poor documentation of ensuing health differences, but in biased findings and ineffective interventions.^12^ Thus, in preparing for future pandemics, it may not be actionable to simply identify target groups without a more nuanced understanding of how inequalities operate. Despite all this, empirical applications of the intersectionality framework in COVID-19 research remain limited.

To date, studies examining intersections of, for example, gender, race, ethnicity and social class have predominantly focused on COVID-19 mortality in the USA.^13^,^16^ Although these categories of social stratification are relevant to health inequalities across various contexts, their configurations are context-specific, with different public health strategies for testing, social distancing and vaccination potentially mitigating or exacerbating their underlying inequalities during the pandemic. For one, unlike most other countries, Sweden did not implement any confinement or mask mandates to combat the spread of the SARS-CoV-2 virus.^17^ This means that despite the country’s past efforts to reduce social inequalities through a strong welfare state, vulnerable groups were potentially more exposed and impacted by the virus than their counterparts in other countries with greater pre-existing health disparities. Yet, while evidence of social inequalities in COVID-19 morbidity and mortality in Sweden by age, gender, migrant background and socioeconomic status exists,^18^,^20^ no study has examined the interacting influence of multiple social factors on these risks. The aim of this study is thus to examine intersectional inequalities in COVID-19 morbidity and mortality in Sweden across groups defined by age, gender, migrant status and socioeconomic position. This will contribute empirical evidence of the intersectional public health consequences of the pandemic within Europe in general, as well as of the unrestrictive pandemic approach of Sweden specifically.

## Materials and methods

### Data and study population

**Figure 1:**
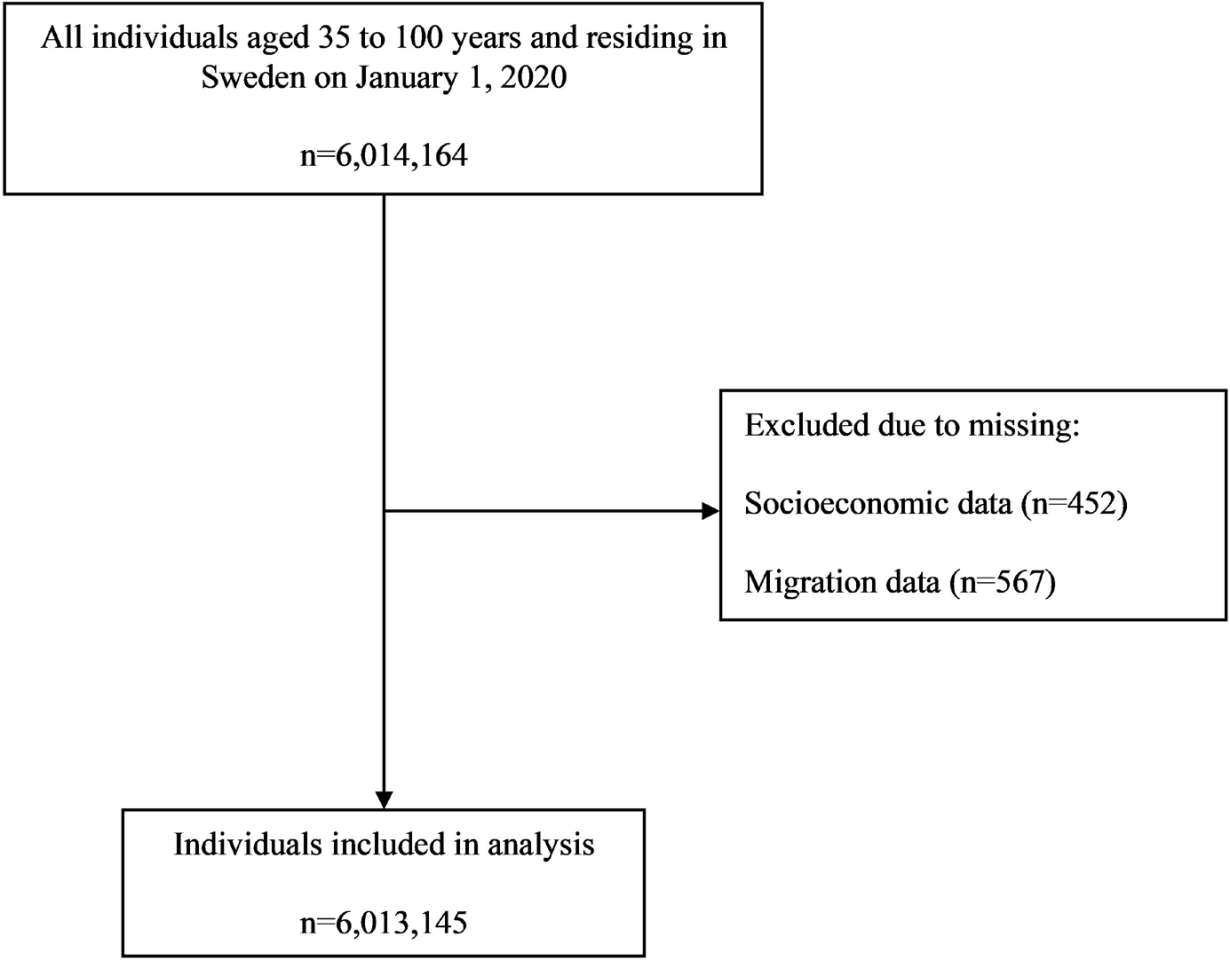
Flow chart for sample selection: Individuals aged 35–100 years and residing in Sweden on 1 January 2020 (n=6 013 145).

This retrospective cohort study was conducted as part of the ‘Explaining COVID-19 mortality among immigrants in Sweden from a social determinants of health perspective’ (COVIS) project.^21^ We followed the Strengthening the Reporting of Observational Studies in Epidemiology guidelines. The study used data from Swedish total population registers linked together using pseudonymised personal identity numbers, including the Total Population Register (TPR) and Longitudinal Integrated Database for Health Insurance and Labour Market Studies (LISA), for information on demographic, socioeconomic and migration-related factors; and the National Patient Register (NPR) and Cause of Death Register (CDR), for hospitalisation and mortality information, respectively. Using the TPR, we identified all individuals registered in Sweden on 1 January 2020 who turned 35 to 100 years old that year (n=6 014 164; see figure 1 for sample selection flow chart). We did not include adults below the age of 35 years due to low event counts. Individuals with missing information on demographic or socioeconomic factors (n=452) or migrant status (including stateless; n=567) were excluded. The final analytical sample consisted of 6 013 145 individuals. Follow-up ended on 31 December 2022.

### Patient and public involvement

No patients nor public were involved in the research.

### Outcomes

We created dichotomous indicators of hospitalisations with a main diagnosis of COVID-19 in the NPR and deaths with an underlying cause of COVID-19 infection in the CDR, identified using the International Classification of Diseases (ICD) 10th Revision emergency codes U07.1 and U07.2. Outcomes occurring prior to the introduction of these new codes in April 2020 were coded retroactively. We additionally identified deaths using the earlier ICD-10 code B34.2, which may have been overlooked during coding.

### Intersectional characteristics

Individuals were divided into intersectional strata based on four variables: age, gender, migrant status and income. Age at baseline was divided into categories of 35–44, 45–54, 55–64, 65–74, 75–84 and 85–100 years old. Gender was dichotomised as men and women, based on the individual’s legal gender. Migrant status was categorised as native-born (Sweden), foreign-born from the Global North and foreign-born from the Global South, categorised using the 2020 Human Development Index (with origin country scores ≥0.800 categorised as Global North, listed in online supplemental table S1).^22^ Individual disposable income (total income from salaries and allowances, minus taxes) at baseline was divided into tertiles (low, medium and high) calculated by age group and gender. Given that income information is reported to the authorities at the end of each year, baseline data were drawn from 2019, and if unavailable, supplemented with 2020 data. Variable operationalisations were motivated by previous evidence of differences in COVID-19 outcomes,^18 19^ and to ensure sufficient events and sample size across intersectional strata. A total of 108 strata (6 age × 2 gender × 3 migrant status × 3 income categories) were included in our analyses. Due to insufficient events within younger age strata, mortality data was only analysed for the population aged 55 years and older, resulting in 72 social strata (4×2×3×3). All strata were assigned a unique identifier (ID) based on a sequence of values, with eg, ID 1111 corresponding to young (aged 35–44 years; age=1) men (gender=1) born in Sweden (migrant status=1) with low income (income=1).

### Statistical analysis

The study applied intersectional multilevel analysis of individual heterogeneity and discriminatory accuracy (MAIHDA) to assess inequalities in COVID-19 disease outcomes across intersectional strata.^23^ Generally speaking, the approach aims to isolate the variance attributable to intersectional groups, as opposed to individual attributes, by nesting individuals (level 1) within intersectional strata (level 2), specifying random effect terms to account for heterogeneity within strata, then comparing the variance terms of empty and adjusted models with additive effects. MAIHDA presents a parsimonious alternative to traditional intersectionality methods, including ‘single-level’ regression approaches with interaction effects (which would require a separate coefficient for every stratum combination) and cross-classification methods (exploring outcome variance across stratified groups), and is well suited to dealing with estimate shrinkage for small strata and when handling rare binary outcomes.^23^,^25^

Following a MAIHDA tutorial by Evans *et al*,^23^ we ran multilevel logistic regression analyses using the ‘melogit’ function, first specifying an empty model with a random intercept for each intersectional stratum (without covariates), followed by an adjusted model including the main effects (ie, individual exposure variables). Next, post-estimation commands were applied to calculate the variance partition coefficient (VPC), or the proportion of total variance that is attributable to differences between strata (in the empty model) and the remaining between-strata variance after adjusting for main effects (which can reflect interactions in the main effects model), taking on a value between 0 and 1.^23^ We then estimated the proportional change in variance (PCV) to indicate the amount of between-stratum variance explained by the addition of main effects as we moved from the empty to the main effects models. A PCV lower than 100% indicates that not all between-stratum variance is explained by the main effects, that is, that the interaction effects are relevant to understanding between-strata inequalities. Finally, the area under the receiver operating characteristic curve (AUC) statistic was estimated to indicate the capacity of the intersectional strata to predict the outcome at the individual level, thus representing a measure of discriminatory accuracy and clinical relevance. The AUC can take on a value between 0.5 (no discrimination) and 1.0 (perfect discrimination), with values above 0.6 generally being considered acceptably discriminatory.^26^ Predicted probabilities and approximate 95% CIs (assuming no sampling covariability between coefficients and random effects) of each COVID-19 outcome were estimated for all intersectional strata. Predicted stratum random effects, or the total predicted probability in a given stratum subtracted by the predicted probability based on additive main effects, were estimated with approximate simulation-based 95% CI. Finally, as a sensitivity analysis, we excluded individuals who emigrated at any time during follow-up. All analyses were conducted using Stata V.19.^27^

## Results

The study population consisted of 6 013 145 individuals aged 35 to 100 years residing in Sweden on 1 January 2020 (table 1). Until 31 December 2022, 77 844 individuals (1.29% of the study population) were hospitalised for COVID-19 and 18 126 (0.30%) died from the disease. Approximately a third of the population was 65 years or older at the beginning of the pandemic (36.13%), and half were men (49.34%). Most individuals were born in Sweden (78.88%), with 11.65% born in the Global North (outside Sweden) and 9.48% in the Global South. The study population was evenly distributed across income tertiles (low: 33.15%; medium: 33.44%; high: 33.41%).

For the full study population, we generated 108 intersectional strata with sample sizes ranging from 84 to 201 749 individuals (see table 2 and online supplemental table S2 for the sample distribution by intersectional stratum). Only three strata consisted of 100 individuals or less (2.78% of all strata), whereas about half had samples of 10 001 to 100 000 individuals. For the older study population included in our mortality analyses, the 72 strata consisted of 84 to 184 774 observations, with a similar sample size distribution as for the full study population.

**Table 1:** Descriptive statistics of the study population residing in Sweden on 1 January 2020

	No. of individuals (%)	COVID-19 outcomes		Emigrations (%)	Deaths due to other causes (%)
		Hospitalisations (%)	Deaths (%)		
Total	6 013 145	77 844 (1.29)	18 126 (0.30)	49 686 (0.83)	260 179 (4.33)
Age (at baseline)					
35–44 years	1 289 202 (21.44)	5621 (0.44)	69 (0.01)	25 092 (1.95)	2741 (0.21)
45–54 years	1 335 769 (22.21)	10 156 (0.76)	286 (0.02)	12 049 (0.90)	6703 (0.50)
55–64 years	1 215 650 (20.22)	13 753 (1.13)	802 (0.07)	7051 (0.58)	17 072 (1.40)
65–74 years	1 101 543 (18.32)	15 805 (1.43)	2455 (0.22)	3693 (0.34)	44 193 (4.01)
75–84 years	766 177 (12.74)	19 689 (2.57)	5979 (0.78)	1464 (0.19)	83 268 (10.87)
85–100 years	304 804 (5.07)	12 820 (4.21)	8535 (2.80)	337 (0.11)	106 202 (34.84)
Gender					
Men	2 967 141 (49.34)	45 442 (1.53)	10 164 (0.34)	28 880 (0.97)	129 730 (4.37)
Women	3 046 004 (50.66)	32 402 (1.06)	7962 (0.26)	20 806 (0.68)	130 449 (4.28)
Migrant status					
Native-born	4 743 001 (78.88)	53 200 (1.12)	14 429 (0.30)	11 146 (0.23)	225 610 (4.76)
Foreign-born, Global North	700 349 (11.65)	12 522 (1.79)	2627 (0.38)	22 739 (3.25)	28 887 (4.12)
Foreign-born, Global South	569 795 (9.48)	12 122 (2.13)	1070 (0.19)	15 801 (2.77)	5682 (1.00)
Income (tertiles by age/gender)					
Low	1 993 650 (33.15)	32 794 (1.64)	7103 (0.36)	34 510 (1.73)	104 584 (5.25)
Medium	2 010 525 (33.44)	24 583 (1.22)	6208 (0.31)	5580 (0.28)	87 402 (4.35)
High	2 008 970 (33.41)	20 467 (1.02)	4815 (0.24)	9596 (0.48)	68 193 (3.39)

**Table 2:** Sample size of intersectional strata for main and subgroup analyses

Strata sample size	No. of strata (% of all strata)	
	Total adult population (108 strata; n=6 013 145)	Older adult population (55+ years) (72 strata; n=3 388 174)
1–100	3 (2.78)	3 (4.17)
101–1000	4 (3.70)	4 (5.56)
1001–10 000	19 (17.59)	18 (25.00)
10 001–100 000	53 (49.07)	30 (41.67)
100 001–500 000	29 (26.85)	17 (23.61)

In the multilevel logistic regression analyses, we found that 23.52% of the variance (VPC) in COVID-19 hospitalisations lay between intersectional strata in the empty model (table 3). With the addition of main effects, all of which were significant—with higher odds of hospitalisation by increasing age and migrant status, but lower odds for women and those with higher income—the VPC decreased to 0.60% (PCV=98.0%), indicative of minimal residual multiplicative effects of the intersectional strata. The empty and main effects AUC of 0.728 suggested that the models have an acceptable discriminatory accuracy in identifying individuals at risk of COVID-19-related hospitalisation based on their intersectional strata. For COVID-19 mortality among older adults aged 55 years and above, we observed 45.50% of the variance between strata, which decreased to 2.22% with the addition of main effects (PCV=97.3%). All main effects were significant and followed the same patterns as in the previous analyses. The residual variance suggested the presence of multiplicative, intersectional health effects, with a considerably high AUC of 0.835.

**Table 3:** Multilevel logistic regression results of COVID-19 hospitalisations and mortality among adults in Sweden

	Hospitalisations Adults 35–100 years (n=6 013 145)				Mortality Adults 55–100 years (n=3 388 174)			
	Empty model		Main effects model		Empty model		Main effects model	
	OR	95% CI	OR	95% CI	OR	95% CI	OR	95% CI
Fixed effects: Regression coefficients								
Intercept	0.013	0.012 to 0.014	0.004	0.004 to 0.005	0.005	0.003 to 0.007	0.001	0.001 to 0.001
Age								
35–44 years			Ref.				–	
45–54 years			1.94	1.76 to 2.15			–	
55–64 years			3.15	2.85 to 3.48			Ref.	
65–74 years			4.33	3.92 to 4.79			3.79	3.06 to 4.71
75–84 years			7.96	7.19 to 8.82			15.49	12.51 to 19.18
85–100 years			13.51	12.17 to 15.01			54.16	43.73 to 67.07
Gender								
Men			Ref.				Ref.	
Women			0.64	0.60 to 0.67			0.48	0.41 to 0.56
Migrant status								
Native-born			Ref.				Ref.	
Foreign-born, Global North			1.71	1.59 to 1.83			1.58	1.33 to 1.86
Foreign-born, Global South			3.28	3.05 to 3.53			2.36	1.95 to 2.85
Income								
Low			Ref.				Ref.	
Medium			0.88	0.82 to 0.94			0.77	0.64 to 0.91
High			0.72	0.67 to 0.78			0.50	0.42 to 0.61
Random effects: Variance								
Stratum-level	1.01	0.87 to 1.17	0.02	0.01 to 0.03	2.75	1.96 to 3.85	0.07	0.05 to 0.12
Summary statistics								
Variance partition coefficient	23.52%		0.60%		45.50%		2.22%	
Proportional change in variance			98.0%				97.3%	
Area under receiver operating characteristic curve	0.728		0.728		0.835		0.835	

**Figure 2:**
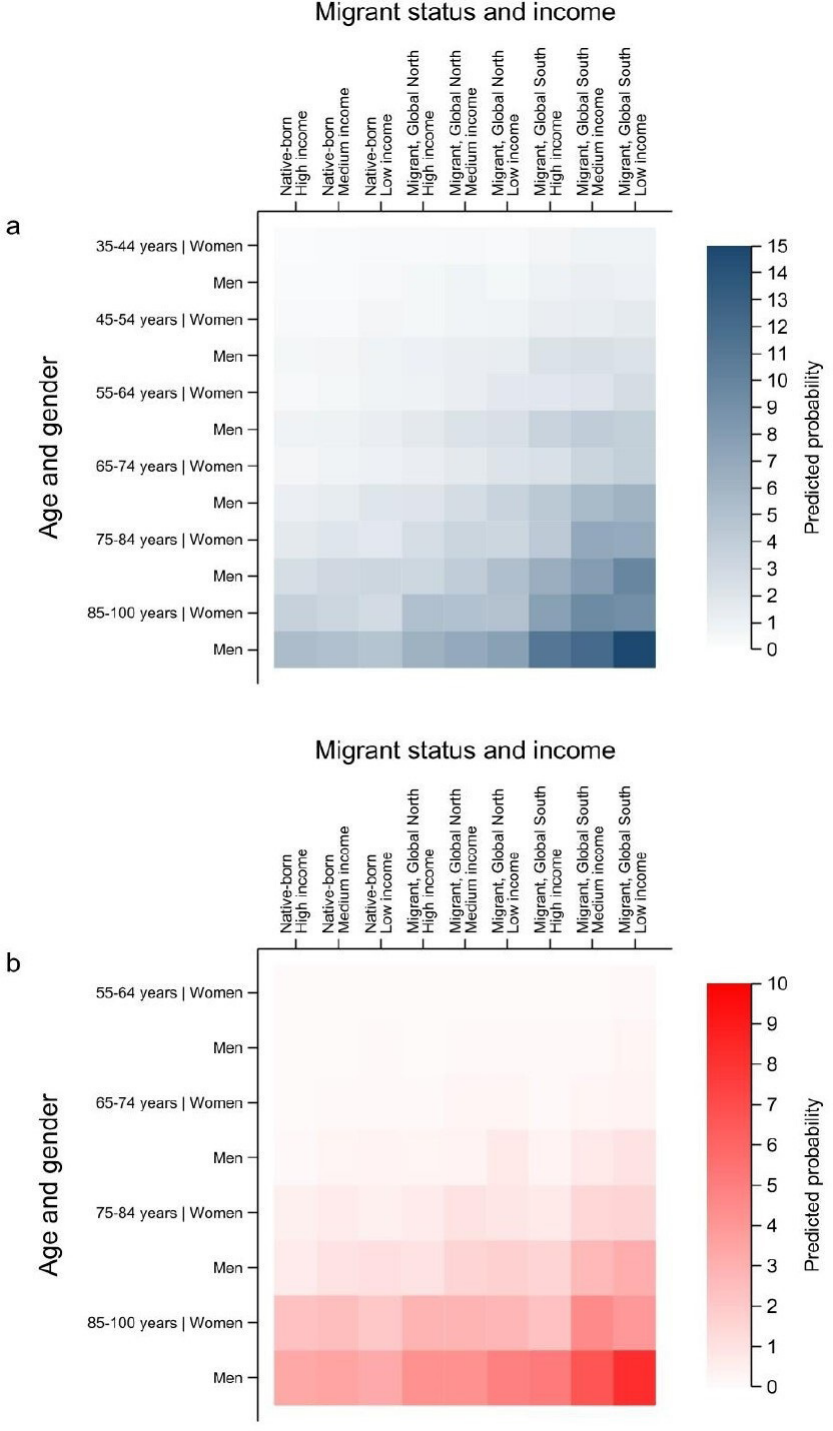
Heatmaps of predicted probabilities by intersectional strata for (**a**) COVID-19 hospitalisations among adults aged 35–100 years and (**b**) COVID-19 mortality among adults aged 55–100 years. Intersectional strata are ordered along the x-axis by migrant status (including region of origin) and income level, and along the y-axis by age group and gender.

Based on predicted probabilities, the youngest (35–44 years) women born in Sweden with high income were the least likely to be hospitalised for COVID-19 (0.19%, 95% CI 0.16% to 0.21%; figure 2, online supplemental table S2 and online supplemental table S3). The oldest (85–100 years) men born in the Global South with low income were the most likely to be hospitalised (14.85%, 95% CI 12.84% to 17.10%). For the older adult subpopulation, predicted probabilities of COVID-19 mortality ranged from 0.01% (95% CI 0.01% to 0.02%) for the youngest included age group (55–64 years) of native-born women with high income to 8.25% (95% CI 6.32% to 10.70%) for the oldest foreign-born men from the Global South with low income. Overall, predicted probabilities of COVID-19 hospitalisations and mortality increased with age, for men and foreign-born (from the Global North, then from the Global South), and decreased with income. For both outcomes, age-based and gender-based inequalities were generally highest among foreign-born from the Global South (eg, for probability differences in hospitalisations, by age: foreign-born men from the Global South with low income=13.80% vs equivalent native-born=4.33%; and by gender: oldest foreign-born from the Global South with low income=5.66% vs equivalent native-born=1.97%), whereas patterns of income inequalities appeared to vary more extensively (see below).

We found intersectional inequalities (ie, significant multiplicative interaction effects) across various strata for both COVID-19 hospitalisations and mortality (online supplemental figure S1 and online supplemental table S2). Most notably, the oldest native-born and foreign-born individuals from the Global North appeared to have narrower income inequalities in both COVID-19 mortality (and among women, hospitalisations) than would be expected with additive effects alone, given the presence of negative interaction effects among those with low income and positive interaction effects among those with high income (eg, for mortality among foreign-born women from the Global North with low income: −0.76, 95% CI −1.37 to −0.11; and high income: 1.07, 95% CI 0.39 to 1.82). For hospitalisations among the youngest low-income groups, we found positive interaction effects in native-born men and women, but negative interaction effects in foreign-born men, suggesting wider income inequalities in the former but narrower in the latter. For mortality, all 55–64-year-old men with low income had positive interaction effects, indicative of wider income inequalities than would be expected.

Sensitivity analyses excluding individuals who emigrated at any time during follow-up did not alter the results (online supplemental table S4).

## Discussion

This study identified inequalities in COVID-19 morbidity and mortality across intersectional groups defined by age, gender, migrant status and income in Sweden with relatively high discriminatory accuracy. Overall, the highest probabilities of experiencing a COVID-19 outcome were concentrated in the oldest foreign-born men from the Global South with low income, and the lowest probabilities in young women born in Sweden with high income. We found evidence of multiplicative intersectional health effects, suggesting that the probability of experiencing COVID-19 hospitalisation or death was significantly altered when multiple social factors were considered simultaneously. Specifically, the effect of income was found to vary across intersectional groups—income inequalities in COVID-19 hospitalisations were most apparent in younger native-born groups, whereas both native-born and foreign-born men had wider income inequalities in mortality, all compared with the oldest groups.

The study has many strengths, including its use of robust longitudinal total population data and its application of MAIHDA, hailed as the ‘gold standard’ of quantitative intersectionality methods.^23^ Together, these strengths allowed us to examine variation in two relatively rare outcomes across multiple, discrete intersectional strata, as well as to identify multiplicative effects nested at the intersection of social groups. Even with total population data, the nature of intersectionality approaches means that some strata will have relatively small sample sizes, which may be especially problematic with rare outcomes. MAIHDA is well suited to dealing with estimate shrinkage due to small strata samples,^24 25^ providing more conservative estimates.^23^ Given that our smallest strata appeared to have some of the highest outcome probabilities (eg, the oldest foreign-born men from the Global South), we may have even underestimated the intersectional differences in our outcomes. Similarly, our choice to restrict the sample to adults aged 35 years or older (for hospitalisations) and 55 years or older (for mortality) may have masked some of the lowest probabilities in the total population, further contributing to a conservative interpretation of intersectional inequalities. However, due to these small samples, we were unable to assess potential variability in intersectional inequalities across specific pandemic waves (eg, after vaccine roll-outs).

In terms of limitations, longitudinal extensions of MAIHDA, particularly for survival analysis, are still lacking.^23^ Yet, our choice to use a standard multilevel logistic regression approach was unlikely to significantly alter the results, given the brevity of our study’s follow-up and the lack of bias from emigrations in our sensitivity analyses. The use of administrative data also presented some limitations, as we were unable to capture information on individuals’ race or ethnicity, both factors relevant to intersectionality research in general and for evidence on COVID-19 outcomes specifically, especially given the potential role of structural racism and discrimination.^4^ Furthermore, while we reported legal or state-recognised gender, which the individual can apply to change from their gender assigned at birth, we could not capture trans or non-binary individuals in the data.

Beyond supporting previous evidence of increased COVID-19-related risks by older age^2^ and for men,^28^ foreign-born individuals^3^ and individuals with low socioeconomic status,^5^ our study confirms the presence of inequalities across multiple intersecting social dimensions. In the USA, research has revealed significantly higher COVID-19 mortality rates among men from vulnerable groups, including racial-ethnic minorities,^13 14^ undocumented immigrants,^14^ and those with low socioeconomic position,^14 15^ supporting our finding of the highest probabilities of both COVID-19 mortality and hospitalisations among low-income foreign-born men from the Global South. Most of these studies present age-adjusted results, making it difficult to directly compare findings across discrete age groups. Outside of the US, evidence on intersectional inequalities is limited, with only one relevant study from Switzerland examining the distribution of risks from COVID-19 testing to mortality using interaction terms based on age, sex and socioeconomic categories.^29^ It found that women had consistently lower risks of COVID-19-related hospitalisation and death than men across all age groups, with greater gender-based inequalities by low socioeconomic status, aligning with our results. We additionally found greater age- and gender-based inequalities by migrant status (which the previous study did not examine),^29^ with evidence of income inequalities in COVID-19 risks varying by age, gender and migrant status.

While we cannot pinpoint the specific mechanisms underlying the complex inequalities in COVID-19 morbidity and mortality revealed in our study, they are likely to result from a combination of mechanisms related to differential exposure to the virus, through employment and housing conditions; differential susceptibility to the disease, through underlying comorbidities; and differential response to the disease, through healthcare-seeking and provision.^17 21^ Our approach specifically suggests that these disadvantages may be unequally distributed across intersectional groups. First, low-income and foreign-born groups are more likely to work in high-exposure frontline occupations and reside in overcrowded housing,^3 5 8^ supporting our finding of their high COVID-19-related risks and the wider income-related inequalities in the working-age population. Second, susceptibility clearly increases with age, yet its role across other social dimensions is less evident. For instance, foreign-born individuals, including men, have been found to have specific morbidity and mortality advantages that would in fact imply a decreased susceptibility to severe COVID-19 outcomes,^7 8^ suggesting that other mechanisms may be more relevant. Regarding the final mechanism, vulnerable groups are less likely than the general population to seek care when needed, or may face discriminatory behaviours in terms of delayed access from the provider side, potentially resulting in more severe outcomes.^30^ While we found evidence of both higher treatment (ie, hospitalisation) and mortality risks among these groups, it is possible that they experienced unequal access to necessary care earlier in the treatment pathway.

Our intersectional approach showed relatively high discriminatory accuracy, and adding other social factors, such as race and ethnicity, could have increased this accuracy further while identifying more ‘at-risk’ groups. Yet, clinicians should interpret these findings with caution,^26^ given that they largely reflect the contribution of age, the most important determinant of severe COVID-19 disease and mortality.^2^ The study remains highly relevant from a public health perspective, advocating for universal prevention measures including mask mandates and lockdowns to protect vulnerable groups that are difficult to identify or reach in a rapidly developing situation such as a pandemic. It also supports the ongoing need to reduce modifiable (eg, income-based) social inequalities to mitigate the potential unequal impacts of future pandemics.

To our knowledge, ours is the first COVID-19 study in Europe to adopt an intersectional perspective including migrant status. This is especially relevant in Sweden, where the higher COVID-19 morbidity and mortality among migrants sharply contrasts with their general health advantages observed before the pandemic.^7 8^ The Swedish context also remains a pertinent case study of how inequalities were influenced by the lax pandemic approach. Future research should consider the lived experiences of the vulnerable groups most affected by the pandemic and its accompanying response in Sweden, to identify underlying mechanisms and the role of intersecting dimensions of disadvantage. Our study should additionally be replicated in international settings with greater pandemic restrictions to provide policy-relevant insights into how intersectional inequalities in health were impacted.

## Supplementary material

10.1136/bmjph-2025-004719 online supplemental file 1

10.1136/bmjph-2025-004719 online supplemental file 2

## Data Availability

Data may be obtained from a third party and are not publicly available.
