# Supplementary material for: Intersectional inequalities in COVID-19 morbidity and mortality in Sweden: a retrospective population-based cohort study
Source: BMJ Public Health. 2026 Apr 10;4(2):e004719. doi: 10.1136/bmjph-2025-004719 (PMC13084887; doi:10.1136/bmjph-2025-004719)
Supplement: online supplemental file 1 [file bmjph-4-2-s001.docx]

**SUPPLEMENTARY MATERIALS**

**Supplementary Table S1. List of Global North Countries (based on 2020 Human Development Index scores of 0.800 or above); all unlisted countries considered Global South**

| Global North Countries | |
| --- | --- |
| Andorra | Latvia |
| Antigua and Barbuda | Liechtenstein |
| Argentina | Lithuania |
| Australia | Luxembourg |
| Austria | Malaysia |
| Bahrain | Malta |
| Barbados | Montenegro |
| Belarus | Netherlands |
| Belgium | New Zealand |
| Brunei Darussalam | Norway |
| Bulgaria | Oman |
| Canada | Panama |
| Chile | Poland |
| Costa Rica | Portugal |
| Croatia | Qatar |
| Cyprus | Romania |
| Czechia | Russian Federation |
| Denmark | Saint Kitts and Nevis |
| Estonia | San Marino |
| Finland | Saudi Arabia |
| France | Serbia |
| Georgia | Seychelles |
| Germany | Singapore |
| Greece | Slovakia |
| Hong Kong, China (SAR) | Slovenia |
| Hungary | Spain |
| Iceland | Sweden |
| Ireland | Switzerland |
| Israel | Trinidad and Tobago |
| Italy | Türkiye |
| Japan | United Arab Emirates |
| Kazakhstan | United Kingdom |
| Korea (Republic of) | United States |
| Kuwait | Uruguay |

**Note:** Monaco is not included in the Human Development Index (HDI), but is considered part of the Global North for analytical purposes. Former states, including Czechoslovakia, Malaya, the Soviet Union, Serbia and Montenegro, and Yugoslavia, are considered part of the Global North based on a majority membership of their comprised current states.

**Supplementary Table S2. List of all intersectional strata and their predicted probabilities and interaction effects for COVID-19 hospitalizations and mortality** (See Excel)

**Supplementary Table S3. Lowest and highest predicted probabilities of COVID-19 hospitalizations and mortality, ranked by intersectional stratum**

| **A. Hospitalizations** (adults aged 35-100 years) | | | | | | | | |
| --- | --- | --- | --- | --- | --- | --- | --- | --- |
| **Rank** | **ID** | **Age** | **Gender** | **Migrant status** | **Income** | **N** | **Prob.** | **95% CI** |
| 1 | 1213 | 35-44 years | Women | Native-born | High | 168,215 | 0.19 | 0.16-0.21 |
| 2 | 1212 | 35-44 years | Women | Native-born | Medium | 156,575 | 0.24 | 0.21-0.27 |
| 3 | 1113 | 35-44 years | Men | Native-born | High | 178,495 | 0.28 | 0.25-0.32 |
| 4 | 1112 | 35-44 years | Men | Native-born | Medium | 162,629 | 0.30 | 0.26-0.34 |
| 5 | 2213 | 45-54 years | Women | Native-born | High | 189,506 | 0.31 | 0.27-0.35 |
| 104 | 6232 | 85-100 years | Women | Foreign-born, Global South | Medium | 943 | 9.48 | 7.89-11.34 |
| 105 | 5131 | 75-84 years | Men | Foreign-born, Global South | Low | 5,776 | 9.81 | 8.74-11.01 |
| 106 | 6133 | 85-100 years | Men | Foreign-born, Global South | High | 91 | 11.18 | 8.72-14.23 |
| 107 | 6132 | 85-100 years | Men | Foreign-born, Global South | Medium | 84 | 12.18 | 9.53-15.44 |
| 108 | 6131 | 85-100 years | Men | Foreign-born, Global South | Low | 1,306 | 14.85 | 12.84-17.10 |
| **B. Mortality** (adults aged 55-100 years) | | | | | | | | |
| **Rank** | **ID** | **Age** | **Gender** | **Migrant status** | **Income** | **N** | **Prob.** | **95% CI** |
| 1 | 3213 | 55-64 years | Women | Native-born | High | 176,311 | 0.01 | 0.01-0.02 |
| 2 | 3212 | 55-64 years | Women | Native-born | Medium | 167,993 | 0.02 | 0.02-0.03 |
| 3 | 3223 | 55-64 years | Women | Foreign-born, Global North | High | 18,060 | 0.03 | 0.02-0.04 |
| 4 | 3113 | 55-64 years | Men | Native-born | High | 184,774 | 0.04 | 0.03-0.05 |
| 5 | 3222 | 55-64 years | Women | Foreign-born, Global North | Medium | 21,550 | 0.04 | 0.03-0.07 |
| 68 | 6232 | 85-100 years | Women | Foreign-born, Global South | Medium | 943 | 4.55 | 3.23-6.36 |
| 69 | 6121 | 85-100 years | Men | Foreign-born, Global North | Low | 4,374 | 4.95 | 3.91-6.26 |
| 70 | 6133 | 85-100 years | Men | Foreign-born, Global South | High | 91 | 5.11 | 3.09-8.35 |
| 71 | 6132 | 85-100 years | Men | Foreign-born, Global South | Medium | 84 | 6.63 | 4.09-10.58 |
| 72 | 6131 | 85-100 years | Men | Foreign-born, Global South | Low | 1,306 | 8.25 | 6.32-10.70 |

**Supplementary Figure S1. Significant predicted stratum interaction effects (difference in total predicted probability in stratum and probability based on additive main effects) for (a) COVID-19 hospitalizations and (b) mortality, ranked low to high by intersectional stratum and labeled by stratum ID.**

**Note: Strata descriptors for (a) COVID-19 hospitalizations, top five negative interaction effects:** 6221 (85-100-year-old foreign-born women from the Global North with low income), 5123 (75-84-year-old foreign-born men from the Global North with high income), 5122 (75-84-year-old foreign-born men from the Global North with medium income), 6211 (85-100-year-old native-born women with low income), 1131 (35-44-year-old foreign-born men from the Global South with low income); **and top five positive interaction effects:** 6113 (85-100-year-old native-born men with high income), 5232 (75-84-year-old foreign-born women from the Global South with medium income), 6213 (85-100-year-old native-born women with high income), 6223 (85-100-year-old foreign-born women from the Global North with high income), 4121 (65-74-year-old foreign-born men from the Global North with low income); **and for (b) COVID-19 mortality, top five negative interaction effects:** 6121 (85-100-year-old foreign-born men from the Global North with low income), 6111 (85-100-year-old native-born men with low income), 6122 (85-100-year-old foreign-born men from the Global North with medium income), 6221 (85-100-year-old foreign-born women from the Global North with low income), 5211 (75-84-year-old native-born women with low income); **and top five positive interaction effects:** 6213 (85-100-year-old native-born women with high income), 6223 (85-100-year-old foreign-born women from the Global North with high income), 6113 (85-100-year-old native-born men with high income), 6212 (85-100-year-old native-born women with medium income), 4121 (65-74-year-old foreign-born men from the Global North with low income).

**Supplementary Table S4. Sensitivity analysis: Multilevel logistic regression excluding individuals who emigrated prior to end of study follow-up**

|  | A. Hospitalizations  Adults 35-100 years (n=5,963,459) | | | | B. Mortality  Adults 55-100 years (n=3,375,629) | | | |
| --- | --- | --- | --- | --- | --- | --- | --- | --- |
|  | **Empty model** | | **Main effects model** | | **Empty model** | | **Main effects model** | |
|  | **OR** | **95% CI** | **OR** | **95% CI** | **OR** | **95% CI** | **OR** | **95% CI** |
| **Fixed effects: Regression coefficients** |  |  |  |  |  |  |  |  |
| ***Intercept*** | 0.013 | 0.013-0.014 | 0.004 | 0.004-0.005 | 0.005 | 0.003-0.007 | 0.001 | 0.001-0.001 |
| ***Age*** |  |  |  |  |  |  |  |  |
| 35-44 years |  |  | Ref. |  |  |  | - |  |
| 45-54 years |  |  | 1.92 | 1.74-2.12 |  |  | - |  |
| 55-64 years |  |  | 3.10 | 2.81-3.42 |  |  | Ref. |  |
| 65-74 years |  |  | 4.25 | 3.85-4.70 |  |  | 3.78 | 3.04-4.69 |
| 75-84 years |  |  | 7.81 | 7.05-8.64 |  |  | 15.40 | 12.44-19.08 |
| 85-100 years |  |  | 13.27 | 11.96-14.73 |  |  | 53.89 | 43.50-66.76 |
| ***Gender*** |  |  |  |  |  |  |  |  |
| Men |  |  | Ref. |  |  |  | Ref. |  |
| Women |  |  | 0.63 | 0.60-0.67 |  |  | 0.48 | 0.41-0.56 |
| ***Migrant status*** |  |  |  |  |  |  |  |  |
| Native-born |  |  | Ref. |  |  |  | Ref. |  |
| Foreign-born, Global North |  |  | 1.74 | 1.62-1.86 |  |  | 1.60 | 1.35-1.89 |
| Foreign-born, Global South |  |  | 3.32 | 3.09-3.56 |  |  | 2.38 | 1.97-2.88 |
| ***Income*** |  |  |  |  |  |  |  |  |
| Low |  |  | Ref. |  |  |  | Ref. |  |
| Medium |  |  | 0.87 | 0.81-0.93 |  |  | 0.76 | 0.64-0.90 |
| High |  |  | 0.71 | 0.67-0.77 |  |  | 0.50 | 0.41-0.60 |
| **Random effects: Variance** |  |  |  |  |  |  |  |  |
| Stratum-level | 1.01 | 0.91-1.13 | 0.02 | 0.01-0.03 | 2.75 | 1.96-3.85 | 0.08 | 0.05-0.12 |
| **Summary statistics** |  |  |  |  |  |  |  |  |
| Variance Partition Coefficient (VPC) | 23.55% |  | 0.59% |  | 45.50% |  | 2.23% |  |
| Proportional Change in Variance (PCV) |  |  | 98.1% |  |  |  | 97.3% |  |
| Area Under Receiver Operating Characteristic Curve (AUC) | 0.728 |  | 0.728 |  | 0.835 |  | 0.835 |  |
